# Living with consequences of stroke and risk factors for unhealthy diet- experiences among stroke survivors and caregivers in Nairobi, Kenya

**DOI:** 10.1186/s12889-021-10522-4

**Published:** 2021-03-16

**Authors:** M. Elmberg Sjöholm, G. Eriksson, A. Bii, J. Asungu, L. von Koch, S. Guidetti

**Affiliations:** 1 Department of Biosciences and Nutrition, Karolinska Institutet, Stockholm, Sweden; 2 Division of Occupational Therapy, Department of Neurobiology, Care Sciences and Society, Karolinska Institutet, Box 23 200, S-141 83 Huddinge, Sweden; 3 Department of Neuroscience, Rehabilitation Medicine, Uppsala University, Uppsala, Sweden; 4 Kenya Medical Training College (KMTC), Nairobi, Kenya; 5 Kenyatta National Hospital, Nairobi, Kenya; 6 Division of Family Medicine and Primary Care, Department of Neurobiology Care Sciences and Society, Karolinska Institutet, Stockholm, Sweden

**Keywords:** Stroke, Risk factors, Nutritional status, Therapy, Rehabilitation, Africa, Caregivers

## Abstract

**Background:**

Stroke prevalence is increasing in sub-Saharan Africa and has been partly attributed to the rapid economic and population growth that have contributed to changes in lifestyle and increased the prevalence of modifiable risk factors for stroke. Healthy diet is important in stroke management and secondary stroke prevention. The aim was to explore the clinical characteristics and functioning after stroke and the experiences of nutritional aspects among stroke survivors and caregivers in Nairobi, Kenya.

**Methods:**

A cross-sectional study with qualitative and quantitative methods involving two rounds of data collection was utilised. In the first round, data on demographics and clinical characteristics were collected for 30 people poststroke during a seminar organized by the Kenya Stroke Association. In the second round, nine participants then agreed to be interviewed together with their caregivers and asked to describe their own experiences and their household eating patterns after suffering a stroke. The food frequency questionnaire and anthropometric measurements of weight, height and waist measurements were used. The self-reported data were analyzed using descriptive statistics and the transcribed interview texts used a constructivist-based theory.

**Results:**

The results give an insight in the life situation for people living with consequences after stroke and their caregivers in Nairobi. The participants were aware that they were overweight and that this indicated an increased risk for the development of cardiovascular diseases. A core category emerged: *The caregiver as the main definer of health and enabler of healthy diet among persons who have had a stroke.* Healthy diets and provided information on eating healthy were lacking from the healthcare professionals, whereupon the responsibility for managing a healthy diet had shifted to the caregivers.

**Conclusions:**

Support needs to be given to people with stroke and their caregivers to achieve a healthy diet. The importance of healthy eating as a way of reducing the risk of suffering a stroke needs to be communicated by health care. The Kenyan food-based dietary guidelines need to be more implemented and accessible as well as an overall secondary stroke prevention program.

**Supplementary Information:**

The online version contains supplementary material available at 10.1186/s12889-021-10522-4.

## Background

Stroke, a cerebrovascular disease, is a non-communicable disease that poses a global challenge [1]. According to the World Health Organization (WHO), low- and middle-income countries (LMIC) bear the heaviest (86%) global stroke burden [1]. African countries are undergoing lifestyle changes, resulting in increased risk factors and burden of stroke. In Kenya non-communicable diseases (NCDs) account for 27% of the mortality and there is often limited access to healthcare due to, poor infrastructure, insufficient numbers of healthcare professionals, as well as the varying socio-economic situation of the population [2, 3]. The proportion of high-risk persons receiving any drug therapy and counselling to prevent heart attacks and strokes is 6% [4] but there are no national guidelines available to guide clinicians on stroke care [5].

Consequences of stroke often entails changes for the person and the family [6, 7] impairments may limit people’s ability to feed themselves after stroke affecting the quality of life and render a poor nutritional status [2]. Dysphagia following a stroke is present amongst 19–81% [8] causing malnutrition, dehydration, and increased mortality [9]. Yet malnutrition is considered a preventable complication after stroke [2, 8]. The impairments after a stroke and the recovery may affect nutritional requirements [10] and nutritional interventions are of importance in managing secondary stroke risk factors [10]. However, as most research and evidence concerning such risk factors originates in high-income countries (HIC), there is a lack of knowledge in the context LMIC and of sub-Saharan Africa (SSA) [8].

Risk factors for stroke are divided into modifiable and non-modifiable risk factors [11]. Modifiable risk factors are susceptible to primary and secondary prevention provided a well-functioning healthcare and support system is in place [9]. Hypertension is considered the single most important risk factor for stroke prevention and is intertwined with excess body weight [12]. Healthy diet entails eating a variety of foods that provide the necessary nutrients to maintain a stable energy level and health. Healthy diet in combination with physical activity and a healthy weight are important contributors to health [1]. Furthermore, according to WHO a healthy diet protects against malnutrition in addition to NCDs such as diabetes, heart disease, stroke and cancer [1].

Kenya is classified as a low middle-income food-deficit country with a weak score on the WHO’s Nutrition Governance Scale [3]. The country suffers the double burden of malnutrition, suggesting undernutrition in the population as well as increasing over-nutrition due to rapid socio-demographic and lifestyle changes [8]. In a country where there are many nutrition issues, there is a great need to develop and scale up nutrition and care packages [3]. An epidemiological transition is taking place in several African countries, including Kenya. The transition is partly attributable to changes in lifestyle [3, 8] resulting in an increased prevalence of NCDs, including stroke [1]. As preventive interventions are considered to be the most effective method of reducing the burden of stroke on a societal level, it is important to understand how the risk factors apply in a LIC country such as Kenya [8]. There is a knowledge gap in how to develop and implement interventions aimed at primary and secondary prevention and the management of stroke [1, 3]. A prerequisite for the development of secondary prevention programmes is knowledge about how people who have had a stroke perceive and experience eating and a healthy diet and the nutritional aspects of living with the consequences of stroke [7, 9]. The aim was to explore the clinical characteristics and functioning after stroke and the experiences of nutritional aspects amongst stroke survivors and caregivers in Nairobi, Kenya.

## Methods

### Design

A cross sectional study using qualitative and quantitative methods involving two rounds of data collection.

### Study setting and participants

In November 2018, the Stroke Association of Kenya (SAK) invited their members (i.e., persons who had had a stroke, caregivers, spouses, parents, children, siblings and/or friends) to participate in a half day seminar about stroke in a location in Nairobi organized by SAK. The invitation also included information about the possibility of participating in a study about living in Kenya after suffering a stroke. The inclusion criteria for the participants in the study were: 1) stroke diagnosis 2) no psychiatric diagnosis, 3) ability to comprehend and reply to instructions in English or in the local language.

After the presentation within the seminar, all people with stroke living in the community that attended the seminar were provided written and verbal information about the study and agreed to participate (*n* = 30) in the ***first round of data collection***. All the participants were members of the SAK and participated voluntarily.

The persons with stroke taking part in the first round, were also asked if they would consider participating together with their caregivers in an additional semi-structured interview (***the second round of data collection***) focusing on experiences of the nutritional aspects of living with the consequences of stroke. Those participants who were interested provided their telephone numbers and indicated their willingness to be contacted for a second round of data collection. The interview would take place at a location that was convenient for them.

### Data collection

In the ***first round of data collection*** persons with stroke (*n* = 30) were interviewed individually by students from Kenya Medical Training College (KMTC) in Nairobi who had been trained for the purpose by the authors SG and LvK. During the interviews, a self-reported questionnaire developed by the authors (LvK, GE, SG) and previously used in Uganda was used to collect the sociodemographic data including age, sex, marital status, handedness, number of children, work status, level of education and housing. Also information on type of stroke, affected side of the body, medication for hypertension and rehabilitation received was asked for during the interview by the person with stroke which also was confirmed by the present significant others (see Supplementary material 1). The Barthel Index (BI) was used to establish the level of dependence in activities of daily living (ADL) [13]. The persons ‘perceptions of the impact of stroke was collected using the Stroke Impact Scale (SIS) 3.0 Uganda version [14]. The scale includes eight domains of functioning: Strength, Hand function, ADL/Instrumental activities of daily living (IADLs), Mobility, Communication, Emotion, Memory and Thinking, and Participation. The SIS aggregated scores range from 0 (indicating more problems in everyday life) to 100 (indicating fewer problems). The SIS 3.0 Uganda version also includes one item to assess the participant’s perceived overall stroke recovery to be reported on a visual analogue scale ranging from 0 = no recovery to 100 = full recovery.

For ***the second round of data collection*** conducted in February – March 2019, the first author (ME) contacted the participants who had provided their telephone numbers during the first round of data collection. They were additionally informed about the study and asked if they were willing to participate in an interview that would take approximately 60–90 min. Nine persons who had had a stroke agreed to participate and the interviews took place in their homes or at the campus of KMTC. Six of the nine participants were accompanied by their caregivers that also participated in the interviews. Therefore, both verbal and written consent was sought from both the person with stroke and their accompanying caregivers. A semi-structured interview was conducted by the first author that focused on the experience and nutritional aspects of living with the consequences of stroke in Kenya (see Supplementary material 2) as well as anthropometric measurements and data on type and frequency of food consumption were collected. Those persons travelling to KMTC received reimbursement for their travelling expenses.

Anthropometric measurements of height, weight, and waist circumference (WC) of the person with stroke were collected. Height was measured using a steel tape attached to a flat wall. The person with stroke was asked to stand to the best of their ability on a flat surface against the wall. The measurement was taken at a point perpendicular to the head, indicated by a wooden block, to the nearest 0.1 cm. Weight was measured to the nearest 0.1 kg using the BMI Beurer SR BF2 scale and BMI indicating normal weight of 18.5–24.9, overweight of 25–29.9 and obese weight over 30 [15]. WC was measured to the nearest 0.1 cm using a tape measure. The risk of developing cardiovascular disease (CVD) increases with a WC of over 88 cm in women and 102 cm in men [15]. Social and demographic data and data on risk factors for stroke and eating habits were collected. To obtain information and guide the qualitative interview on the type and frequency of food consumed, a food frequency questionnaire (FFQ) from the Kenya National Clinical Nutrition and Dietetics Reference Manual was used [16]. In order to capture the participants’ experiences and perspectives, an interview guide that provided guidance on questions regarding the pathway from stroke onset to the time of the interview, the consequences of stroke in everyday life activities, nutritional status and eating habits was used. The interviews were digitally recorded and transcribed verbatim.

### Data analysis

To describe the sample regarding demographic and clinical characteristics, functioning, anthropometric measures and perceived impact of stroke descriptive statistics were used. An algorithm [17] was used to produce the SIS aggregated scores in each domain. The qualitative data were analysed by the first author using a constructivist grounded theory (GT) approach [18]. GT uses an inductive approach to data collection that is non-linear and should be seen as flexible guidelines. Accordingly, the analysis was carried out in two phases and, in the first phase, initial coding was conducted using code labels very close to the text. The second phase used a systematic constant comparative method including moving back and forth in the text and to the emerging codes to analyse and continue categorising. Two additional interviews with new participants were added to reach saturation in the categories. Memos describing the findings and reflections from the interviews and emerging categories were written throughout the process. The analysis resulted in one core category and five sub-categories. The qualitative and quantitative findings of this study were then compiled and synthesised, starting with a structure based on the qualitative categories that emerged using GT constructivist analysis [18] and supported by the quantitative data. Throughout the data analysis process, the last author (SG) repeatedly asked important questions to enhance the credibility of the results. To improve the resonance and as a triangulation of the findings [18], the different categories and subcategories were discussed between the first author (ME) and SG. As a final step in the analysis, these results were discussed with all authors, where the third (AB) and the fourth author (JA) represented the Kenyan context of cultural competence, as a form of informal triangulation [19].

## Results

This study focused on describing the characteristics and functioning after stroke for 30 members of SAK and living in Nairobi, Kenya. Furthermore, out of this sample, nine persons experiences of living with the consequences of stroke were explored with a focus on nutritional aspects and eating habits.

### The characteristics and functioning after stroke

The socio demographic data and the clinical characteristics are presented in Table 1.

**Table 1:** Socio demographics of the study participants data collection- round 1 (*n* = 30) and participants in -round 2 (*n* = 9)

Participants with stroke	*n* = 30	(%)	*n* = 9
**Age (years)** Mean (SD)	54 (15.5)	**–**	52
Range	22–77		(26–70)
**Age, at onset of stroke** (years)			
Mean			47
Range			(10–69)
**Gender:** Men	16	53	4
Women	14	47	5
**Members in a household** Mean (SD)	5.8 (2.3)		
**Number of children living at home (< 18 years)** Mean (SD)	3.8 (5.3)		
**Level of education**			
No education	2	6	
Primary school	5	17	
Secondary school	11	37	
Tertiary school	3	10	
University	9	30	
**Housing after stroke**			
Lives in own house	20	69	
Does not live in own house	9	31	
**Marital status now**			
Married, living together	21	70	6
Married, not living together	1	3	3
Single/widow	8	27	
**Uses assistive aids now**	14	48	
**Working now/before stroke**	14/25	56/86	4/
**Source of Livelihood before/now**	24/20	86/69	
**Dependency Barthel Index (BI)**			
Dependency (0–59)	4	14	
Moderate dependency (60–90)	15	52	
Independent (91–100)	10	34	
**Type of stroke**			
Ischemic	15	54	
Hemorrhagic	7	25	
Do not know	6	21	
**Hemisphere**			
Right	15	55	3
Left	11	41	6
Both	1	4	
**Handedness**			
Right	24	80	
Left	6	20	
**Speech productions**			
No aphasia	19	65	
Limited vocabulary	9	31	
Aphasic	1	3	
**Hand motor power**			
Normal strength	9	32	
Motor Impairment	10	36	
Paralysis	9	32	
**Gait**			
Walks 5 m without aid	19	68	
Walks with aid	2	7	
Walks with aid &help of another person	4	14	
Unable to walk	3	11	
**Orientation** (correct for time, place & person)	22	76	
**Diabetes**	12	40	7
**Hypertension**	19	65	9
**Hypertension medication**	17	–	
**Received after stroke**			
Medical health care	26	87	
Rehabilitation	27	90	
Interventions from traditional healer	9	32	
**Modified Rankin Scale** Mean (range)			2.7 (1–4)
**Weight** (kg) Mean (range)			72.8 (61.4–83.4)
**Height** (cm) Mean (range)			164 (148–178.0)
**BMI** (kg/m^2^) Mean (range)			27.1 (24–29.9)
**WC** (cm) Mean (range)			
men (*n* = 4)			96 (88–107)
women (*n* = 5)			101 (90–108)

The impact of stroke assessed by use of SIS 3.0, Uganda version is presented in Table 2.

**Table 2:** The SIS 3.0 aggregate domain scores and self-rated recovery *n* = 29

SIS Domains	Mean domain score	SD	Median domain score	Range domain score
Strength	48.7	22.3	50	12.5–87.5
Memory & thinking	84.1	18.0	92.8	32.1–100
Emotions	63.1	21.5	58.3	30.6–100
Communication	85.2	17.8	92.9	32.1–100
ADL/IADL	79.8	23.9	71.2	0–100
Mobility	67.4	29.7	76.3	0–100
Hand function	46.5	55	50	0–100
Participation	72.1	81.2	50	20.8–100
Stroke recovery	60.5	19.4	55	30–100

### The participants´ experiences and nutritional aspects

The demographics for the nine participants participating in second data collection, including anthropometric measurements, are presented in Table 1, right column. The socio-economic status was mixed, ranging from slum-dwelling participants to owning several properties. However, most of the participants had a low socio-economic status (SES). The persons with stroke all stated that they had been seriously affected by their stroke and had experienced lengthy hospital stays ranging from two weeks to several months. The participants and their households, all followed the Kenyan staple diet according to the FFQ with the majority of participants consuming white maize at least twice a day.

The mean BMI fell in the overweight range and the lowest BMI was 24. The WC of all five women and two men fell within the increased risk of developing CVD [15].

The self-rated data from the nine participants with stroke of which four were males showed that regarding information provided from health professionals, five of the participants had received information on stroke, three of them had receive information on risk factors for stroke, two of them had received information on possible beneficial effects of diet while seven of them had been provided information from the SAK, relatives, friends or from a nutrition student. Eight of the participants had experienced difficulties chewing or swallowing food or drinks at onset and three of them still experienced dysphagia at time of the interview.

They reported that six caregivers were the main facilitator of their healthy diet. In seven out of nine cases, the person with stroke was fully dependent on their caregiver to manage everyday tasks. Caregivers assisted with dressing, walking, taking a bath, going to the bathroom, cooking, feeding and drinking. The caregiver was also the main facilitator of maintaining social relations, procuring information on diet and care, managing the household economy including the food budget, doctors’ appointments, medication, rehabilitation services, as well as school fees for children. The role of the caregiver differed depending on the participant’s gender. The spouse of the male participants was the primary caregiver. None of the spouses of the women were primary caregivers.

After compilation and analysis of the qualitative and quantitative data from the participants who have had a stroke (n = 9) and caregivers (*n* = 6) one core category, and five sub-categories emerged. The core category was: *The caregiver as the main definer of health and enabler of a healthy diet among persons who have had a strok*e. The sub-categories were: (1) *The perspectives on health as central in the recovery process,* (2) *The caregiver as the collector of information on healthy diet*, (3) *The caregiver as the manager of the person’s with strokes limitations in eating* (4) *The caregiver as the manager of the underlying modifiable risk factors*, and (5) *Need of compromised healthy diet due to loss of income.*

#### The core category: the caregiver as the main definer of health and enabler of healthy diet among persons who have had a stroke

The persons with stroke all stated that they had lost their autonomy as a consequence of their stroke, rendering them dependent on a caregiver to manage their everyday activities. The caregiver took charge of the path to recovery after stroke and was responsible for all aspects of eating healthy. Several of the persons with stroke had received information on how to manage underlying risk factors such as hypertension and diabetes through diet prior to the onset of stroke. However, they appeared to underestimate the value of this advice and lacked understanding of the relationship between diet and health outcome. They also appeared to regret not having valued this advice earlier and described a feeling of being a burden to their caregiver.

Most caregivers expressed confusion and a sense of shame that they did not have access to information on how to best adapt a diet to diabetes, hypertension and dysphagia, for example. Many of them had not been informed by the staff at the hospital that the person for whom they were caring had suffered a stroke. Some information had been made available about healthy diet, but several of the caregivers stated that they had not been able to adapt diet and eating due to financial constraints primarily relating to loss of income due to stroke.

#### The perspectives on health as central in the recovery process

Due to the consequences of stroke, the afflicted person lost their autonomy to the extent that the role of the caregiver went beyond helping with everyday tasks. The caregivers’ own opinions and perspectives on health would largely determine what the person who had suffered a stroke did regarding their recovery process. Whether the caregiver held a passive or active view on health as a process proved important to how diet and physical activity were modified. In effect, a passive view regarding how to act in relation to health left the caregivers feeling that they should not interfere, meaning that the person with stroke was bedridden while the caregiver waited for their health to improve.

In the two cases in which the caregiver was employed, the person with stroke had to remain in charge of their own recovery process due to the caregiver’s lack of information about the consequences of stroke. In these two cases, the two women with stroke had a significantly better socio-economic situation. This may have ensured a higher standard of health care thereby ensuring more information about stroke. Both women stated that they had received extensive information from their doctors about stroke and the risk factors for recurrence of stroke. Both women had the same passive view on health and the recovery process, which resulted in them being bedridden for one and nine years, respectively. In eight out of nine interviews, the person with stroke had been left to rest ranging from many months up to several years. One caregiver explained her husband’s condition during this time: *He couldn’t eat, he couldn’t even sit like he is sitting. He was just sleeping, yes […*] *It was a year that he was now down totally.*

The caregivers stated that during the initial phase after stroke, they had made no efforts to achieve eating healthy. Changes in the diet were limited to adjusting food consistency and portion size as well as helping with feeding and drinking when necessary. Several caregivers stated that the condition of the person with stroke deteriorated during this time due to inactivity, weight gain or weight loss. The eventual realisation that the health of their relative would not improve without their active participation in recovery process caused a change in the caregivers’ views on health. The caregivers stated that this change was because they had received information on stroke from a doctor, friend or neighbour, the need for the relative to regain their function due to financial constraints or receiving information on the importance of managing the underlying risk factors for another stroke. One caregiver described how they had received advice on physical activity: *[…] They also told me he should walk, he should walk sometimes, he should do some walking. To reduce his body size. […] [It made] a lot of change.*

The caregivers stated that they felt either shame or regret at not realising this at an earlier stage.

#### The caregiver as the collector of information on healthy diet

Regardless of whether the person affected by stroke had received dietary advice prior to stroke, the responsibility of gathering information on healthy diet was fully transferred to the caregiver after the stroke. Prior to the stroke, five persons with stroke recalled being cautioned or receiving dietary advice from a doctor or dietitian, primarily to manage hypertension and diabetes. Two men had also been strongly advised to stop drinking what was described as a high amount of alcohol. It appears that this information was not communicated to the caregiver who stated that they had very limited knowledge regarding the advice that the person with stroke received prior to the onset of stroke. During the period of hospitalisation, the participants stated that they had received varying degrees of information on healthy eating and the diets role in recovery.

Several caregivers recalled being instructed by a doctor to manage blood sugar levels but without any guidance of how to do so. The caregivers described how they had returned home with the person with a stroke and had minimal knowledge about what and how to feed them. The caregivers stated that this lack of knowledge affected the health of the person with stroke: *It’s like his health is deteriorating because his eating is poor, he doesn’t do anything, he just stays in the house. There’s not much he can do.* They also expressed desperation about their lack of knowledge regarding what to feed the person and how to feed them: *I still don’t know because there’s no one to tell me, there’s no one to teach me anything.*

The caregivers described how they relied heavily on friends, family, church and colleagues for advice and information on who to turn to for help. The group had limited internet access with only one person stating that they relied on the internet as a source of information. Both caregivers and the persons with stroke stated that they had become socially isolated after the stroke. This was described as a loss of social resources that otherwise could have provided valuable knowledge on healthy eating. Consequently, it was stated that social isolation combined with financial constraints caused the caregiver to be largely confined to the home. This restriction was also expressed as a need to remain in immediate proximity to the person with stroke to help feed them.

The caregivers of lower SES stated that they felt uncertain about who to turn to for help and were too afraid to ask doctors or dieticians. Both caregivers and persons with stroke also repeatedly stated that they had difficulty attending doctors’ appointments due to lack of funds for travelling to and from the appointment, as well as paying the doctors’ fees. Several participants also stated how they needed to accept their stroke as a medical condition and that it was not a result of witchcraft. Conversely, the four persons with stroke who had a higher economic status explained how friends and colleagues were a source of direct information.

#### The caregiver as the manager of the person’s with strokes limitations in eating

As support from healthcare institutions for managing dysphagia and limitations to feeding was lacking, the caregiver became responsible for managing these conditions. The caregivers described how they attempted to deal with and navigating these consequences to the best of their ability and tried to compensate for the skills that the person with stroke was lacking instead of finding new ways for the person to perform them.

When the participants were asked whether they were familiar with the term dysphagia, none of them had heard of this term. The caregiver stated that they eventually realised that there was a serious choking hazard that needed to be addressed and that they did so by adapting the consistency of the food*: We could make food and then mash it as for a small child.* The caregiver stated that that food intake of the person with stroke had significantly decreased while they were suffering from dysphagia.

Eight of the persons with stroke stated that they had persistent difficulties in either one or several of the following three areas: feeding themselves, preparing food and grocery shopping: *Like now, cooking ugali […] For the family, that’s a problem. I can’t manage.* One woman stated that she had difficulty preparing ugali, a process that demanded the use of both hands: *Due to my stroke, when it comes to cooking ugali, I am challenged.* Not being able to cook or shop for groceries was described as being problematic by the women with stroke but not by the men, who predominantly stated that they were most affected by their inability to feed themselves. The caregivers received no support from either an occupational therapist or a dietician in how to adapt to these challenges. The lack of support led the caregivers to compensate for any skills that the participant was lacking. When asked if the participant was practicing regaining their lost skills, the caregivers often responded that it was easier for them to perform the task themselves as it was painful to watch the person with stroke struggling.

#### The caregiver as the manager of the underlying modifiable risk factors

All persons with stroke stated that they suffered from modifiable underlying risk factors, putting them at risk of stroke recurrence. This needed to be managed in part through diet and physical activity. Having previously overseen managing these risk factors themselves, the stroke meant that the responsibility shifted to the caregiver.

Before having the stroke, the participant described how they themselves took charge of managing conditions such as diabetes and hypertension. Both the person with stroke and the caregiver believed that they were managing the condition well: *Yes, it has been very well managed. I was surprised, at the time, that there even was a stroke.* It was not until after the stroke that both the participant and the caregiver stated that they realised that it might not have been managed as well as they had believed. Even those participants who had received extensive information on how to manage diabetes, for example, stated that they had not understood the importance of these instructions*: They tried to teach me on nutrition […] they gave me some advice, but I didn’t take it seriously.*

Both the person with stroke and the caregiver stated that they wanted the person with stroke to *reduce their body size and/or body weight.* The person with stroke stated that their body size was a hindrance and a burden: *my body has become too big for me*. Although the person with stroke expressed their desire to lose weight and become more active, they stated that the initiative to achieve change was the responsibility of the caregiver. The caregivers and the two women with employed help described how weight reduction could be achieved by restricting portion size and minimising “overindulgence”. In eight of the interviews, the participants expressed surprise when they learned that there are many foods that must be consumed in moderation. Increasing physical activity was described as a way of reducing body size, although the caregivers stated that this was a challenging task that was often avoided.

All participants and caregivers expressed their surprise that *diabetes* had to be managed so carefully. When describing the major changes that had been made in managing diabetes, two main changes were consistently mentioned: having “brown bread” for breakfast and excluding sugar from traditional Kenyan tea. Again, the person with stroke stated that they did not make these changes on their own but relied on the caregiver to make them. The caregivers stated that it was challenging to monitor the blood glucose level of their relative even though a physician had encouraged them to do so. Two caregivers stated that it took them eight months and 2.5 years, respectively, before they could afford to buy a glucose meter. Once the meter had been bought, the caregivers stated that they struggled with the cost of test strips, which meant that they tested the blood glucose level of the person with stroke once a week or less frequently: *Buying those strips was very expensive because I don’t have money. So now I test maybe once a week*. Knowing that infrequent testing could have consequences, the person with stroke stated that this was a cause for concern: *But when [the testing] she takes that time, I get worried. That’s a problem. I get worried.*

The persons with stroke and the caregivers stated that *hypertension* was managed by excluding or minimising salt intake. All persons with stroke stated that they were prescribed medication to manage hypertension, but the caregivers often stated that adherence to the drug regime was low due to lack of funds: *So I just bought it once and when it was over, I didn’t return for more. […] it was expensive for me.*

The caregiver described how they managed *hypercholesterolemia* by excluding eggs and margarine from the diet. Most persons with stroke were prescribed drugs to manage this condition. However, very few caregivers stated that they were able to provide the drug.

The consequences of stroke shifted the responsibility of managing the underlying risk factors to the caregiver. As they had not been involved in this process prior to stroke, the caregivers stated that managing these conditions was burdensome and stressful as they did not have sufficient information.

#### Need of compromised healthy diet due to loss of income

Loss of income and SES were described as the main reason for the inability to achieve healthy diet. The consequences of stroke caused the loss of either one or two household incomes, putting the caregiver in a position in which they stated that they were forced to compromise between diet, drug regimen and rehabilitation services.

At the time of the interviews, the caregivers and participants all stated that they felt they had acquired knowledge about the changes that needed to be made to the diet of the person with stroke. However, the caregivers stated that their ability to provide this diet was very limited and they were open about how this could be due to a lack of funds. The caregivers generally stated that making changes to the diet was cheaper and easier than visiting different rehabilitation services or taking medication. However, the caregiver stated that being able to afford medication was prioritised in the household budget, putting them in a position in which they felt they had to choose between affording medicine before other items such as groceries, school fees and doctors’ appointments: *I just pay the rent, for most of the little money I buy food. Even that food is not enough*. Often, the caregivers would explain that the person with stroke was supposed to take one tablet of a certain kind of drug per day, for example, but, in reality, the caregiver was only able to provide the medication sporadically: *We just tried once because I told you it’s expensive and I don’t have enough money. I tried for the first two months, then I stopped.* The caregivers stated that the lack of funds caused stress and that they felt that the care they provided was insufficient and could have detrimental effects on the health of the person with stroke.

Most of the caregivers stated that when making changes to the diet, less healthy foods should be replaced with healthier alternatives. The caregivers stated that financial constraints meant that they only excluded and did not replace foods. The caregivers mainly fed the persons with stroke ugali, sweet potatoes and white rice accompanied by vegetables, small amounts of meat and sometimes fruit. Both the persons with stroke and the caregivers stated that red meat was detrimental to their health and was intentionally excluded from the diet. The caregivers stated that they were not able to afford what was described as the healthier alternative – white meat – regularly and that the person with stroke consequently had fewer sources of protein than prior to the stroke.

## Discussion

Stroke burden is increasing in Kenya and several risk factors are lifestyle related and are modifiable through interventions in diet and physical activity [9]. This study aims to explore the clinical characteristics and functioning after stroke and the experiences of nutritional aspects among stroke survivors and caregivers in Nairobi, Kenya. The results from SAK sample give an insight in the life situation for people living with consequences after stroke in Nairobi. The mean age in the sample was much lower than for people who have stroke in HMIC [4]. Furthermore, the majority were moderately dependent in ADL. They perceived an impact after stroke mainly on the SIS domains Strength and Hand function which are similar to findings from research in Uganda [6]. They were less impacted by aphasia, in their orientation and within the SIS domain Memory and thinking and domain Communication. It seems like SAK only reach people who are mainly cognitively intact and without problems with communication.

The results from the second data collection showed that healthy diet was lacking in the study population; pre-existing risk factors were generally insufficiently managed through diet and physical activity; the persons with stroke were overweight; there was a lack of information on healthy diet; and healthcare services providing nutritional support were largely unavailable to the participants. Healthy diet was facilitated through the caregiver who felt that such a responsibility was stressful and burdensome. There are several results that can be discussed but a few key points are described below.

### Lack of nutritional therapies for dysphagia

The persons with stroke stated that they had difficulty feeding themselves, either due to apparent dysphagia, upper extremity paresis or mood changes, for example, depression. A clinical assessment of dysphagia appeared to be lacking even though it is recommended for all stroke patients. Also, dietary modifications have been standardised [10, 18]. In a large review on nutrition in stroke patients, Corrigan et al. found that early detection and treatment of malnutrition – common in Kenya and often a finding in persons with stroke – can affect the person’s ability to participate in rehabilitation and recovery [10]. The apparent lack of clinical assessment of dysphagia in Kenya has been studied and a study assessing the knowledge on stroke care in healthcare professionals in SSA was conducted at one of Kenya’s largest referral hospitals [20]. The study was the first of its kind and the authors conducted a cross-sectional survey of 300 healthcare professionals, of which 199 responded. The findings showed a wide range in knowledge on stroke care and only 25% of responders reported performing dysphagia tests while 30% reported never administering tests [20]. 50% of responders reported documenting a lipid profile of the patients and the same number attended diabetes screening. However, our study had too few participants to determine whether the results correspond with the findings of Lin et al. The experiences of the participants in this study with healthcare professionals were similar to the results of the survey [20]. The authors described the positions held by the responders to the survey and highlighted how stroke care was administered by a single neurologist.

### BMI, WC and overweight

Eight participants with stroke had a BMI indicating overweight. All five women and two men had a WC indicating increased risk for CVD. Overweight is part of the double burden of malnutrition that plagues Kenya and is probably an outcome of various lifestyle factors [4]. In 2012, Mbochi et al. published a study aimed at determining the predictors of overweight and obesity in adult women (*n* = 365) in Nairobi, Kenya and showed that BMI increased with increasing household expenditure [21]. No such study of a male population has been found, possibly because obesity and overweight are more prevalent in women [4, 20]. Although this study is too small to confirm the findings of Mbochi et al., the WCs of all the women put them at risk of CVD. As overweight and obesity are intertwined with other risk factors such as diabetes and hypertension, tackling the increasing prevalence of overweight in the population is perhaps a smart starting point for managing NCDs in Kenya.

### Societal relevance – implications for clinical nutrition in stroke management in Kenya

This albeit small study raises questions that will need to be further explored in order to develop and implement both the national and community-based interventions that are needed to prevent and manage stroke and a healthy diet in Kenya [4]. Kenya is currently one of seven African countries that have developed food-based dietary guidelines, but the lack of nutritional specialists and poor access to healthcare services makes the effect of these guidelines questionable [1, 5]. There is much to learn from how HIC manage NCDs such as stroke. However, there is a need for LMIC such as Kenya to devise their own systems [8]. Unlike many HICs in which these lifestyle changes have occurred over several decades, LMIC countries experience these changes rapidly. Kenya, which has tripled its population over the last 35 years, does not have an extensive healthcare system and knowledge of stroke in the population is low. Thus, developing a national prevention programme poses multiple political and economic challenges [8]. However, this study aims to fill the knowledge gap that will enable policymakers and HCPs to better tailor the secondary prevention of stroke and provide advice on diets and physical activity in a Kenyan and SSA setting.

### Methodological considerations

This study has explored and described a small sample of people living with the consequences of stroke in Nairobi and showed that the structural issues hindering a healthy diet are generalisable to many aspects of health care in LMIC [1, 21]. The participants were all members of the SAK and, at the time of the interviews, had received some information on stroke and risk factors. It is to be assumed that large numbers of people suffer stroke in Kenya, particularly in rural areas, and that these people never get access to the same level of health care or information. Essential healthcare services remain beyond the reach of most Kenyans [3, 22]. If this study were to be repeated with participants who represented different geographical areas of Kenya and who were not members of the SAK, it is possible that eating habits and factors affecting a healthy diet would differ from the results of this study. However, it is not unreasonable to assume that this study population has given a brighter picture of eating habits and the knowledge that exists within the community of people living with the consequences of stroke and their households than what actually exists in a more representative group. The interviews were conducted with English-speaking persons in a country that has two official languages (English and Kiswahili) and 47 ethnic groups. Although the participants represented several different ethnic groups, if the study were to be repeated, it would be of interest to include a wider demographic. Diet varies between ethnic groups and if possible, it would be of great interest to study these variations in relation to the development of stroke and other NCDs.

### Ethical considerations

There have been several ethical considerations for this study even though the participants with stroke and their caregivers participated voluntarily and on their own initiative. Firstly, as stroke caused dramatic changes to the life of the participants and their households, the subject itself is sensitive. Secondly, people living in poverty are often ashamed about their situation. During the interviews, every effort was made to be sensitive to such aspects. As the main point of focus of the interviews was on nutritional aspects, not to value anything else that was said during the interview during the interviews. The reason for this is two-fold: firstly, in order to ensure that the interviewees did not feel they were being judged about their diets, and secondly, to try to avoid the interviewees describing a diet that they believed to be healthy, instead of what they were actually eating. As all information on diet has been self-reported, both under- and overreporting is to be expected. Underreporting often occurs in relation to alcohol consumption which, in Kenya, is frowned on in particular, as well as meat consumption [21]. Many of the interviewees requested individually tailored dietary advice from the first author at the time of the interviews. These persons were encouraged to use some of the information given in the Kenyan Dietary Guidelines. As most of them did not have internet access, the guidelines were sent to the SAK to relay to its members.

## Conclusion

Suffering a stroke in Kenya is a devastating event that often causes long-term disability. Low access to healthcare services in the population, lack of knowledge within the healthcare community and an insufficient number of nutritional specialists all contribute to the knowledge gap that means that individuals must modify the risk factors and manage the consequences of stroke. A healthy diet was lacking in the study population. In order to achieve a healthy diet, more support must be given to caregivers and more work needs to be done in order to communicate the importance of eating healthy as a way of reducing the risk of suffering stroke.

## Supplementary Information

**Additional file 1: Supplementary material 1.** The first round of data collection Description of data included in the file: A self-reported questionnaire developed by the authors (LvK, GE, SG) including sociodemographic data and clinical characteristics as age, gender, marital status, handedness, number of children, work status and level of education and housing, information on type of stroke, side of the body affected by stroke, medication for hypertension and rehabilitation.

**Additional file 2: Supplementary material 2.** The second round of data collection Description of data included in the file: The interview protocol. Not included in the manuscript.

**Additional file 3: Supplementary file 3.** Description of data included in the file: 2020 STROBE_checklist Kenya.

## Data Availability

The data supporting the conclusions of this article are available at the Division of Occupational therapy, Department of Neurobiology Care Sciences and Society, Karolinska Institutet, Stockholm, Sweden. E-mail: susanne.guidetti@ki.se

## Abbreviations

ADL: Activities of daily living
BMI: Body mass index
CVD: Cardiovascular disease
FFQ: Food frequency questionnaire
GT: Grounded theory
HIC: High-income countries
IADLs: Instrumental activities of daily living
KMTC: Kenya Medical Training College
LMIC: Low- and middle-income countries
NCDs: Non-communicable diseases
SES: Socio-economic status
SAK: Stroke Association of Kenya
SIS: Stroke Impact Scale
SSA: Sub-Saharan African
BI: The Barthel Index
WC: Waist circumference
WHO: World Health Organization
