# Supplementary material for: Living with consequences of stroke and risk factors for unhealthy diet- experiences among stroke survivors and caregivers in Nairobi, Kenya
Source: BMC Public Health. 2021 Mar 16;21:511. doi: 10.1186/s12889-021-10522-4 (PMC7968355; doi:10.1186/s12889-021-10522-4)
Supplement: Supplementary file 1 — Additional file 1: Supplementary material 1. The first round of data collection Description of data included in the file: A self-reported questionnaire developed by the authors (LvK, GE, SG) including sociodemographic data and clinical characteristics as age, gender, marital status, handedness, number of children, work status and level of education and housing, information on type of stroke, side of the body affected by stroke, medication for hypertension and rehabilitation. [file 12889_2021_10522_MOESM1_ESM.docx]

***Supplementary material 1***

**Stroke rehabilitation project, Data collection 1.**

**Stroke rehabilitation project Kenya**

**Client-information**

1. Age:…………years
2. Gender Male  (1)

Female  (2)

1. Which side of your body is affected? Right  (1)

Left  (2)

1. Which hand is your dominant hand? (right-handed)  (1)

(left-handed)  (2)

1. Highest level of education No education  (1)

Primary school  (2)

Secondary school  (3)

Tertiary school  (4)

University  (5)

1. What is your religion? Catholic  (1)

Anglican  (2)

Muslim  (3)

Other  (4)

If other, please specify:………………………………………………

**The following questions refer to the everyday life now of persons’ that had had a stroke if other is not specified**

1. a) Do you have a source of livelihood **now?** Yes  (1) No  (2)

b) working yes  (1) no  (2)

c) If yes: full-time  (1)

part-time  (2)

8. Type of work (occupation)

Retired  (1)

Sick pension  (2)

Sick leave  (3)

Unemployed  (4)

Student  (5)

Housewife  (6)

Self-employed  (7)

If self-employed; specify what?....................................................................................

9 a) Did you have a source of livelihood **before stroke?** Yes  (1) No  (2)

b) was working **before stroke?** Yes  (1) No  (2)

c) If yes: full-time  (1)

part-time  (2)

10. Type of work (occupation)

Retired  (1)

Sick pension  (2)

Sick leave  (3)

Unemployed  (4)

Student  (5)

Housewife  (6)

Self-employed  (7)

If self-employed; specify what?..........................................................................

Others  (8)

Others Specify……………………………………

11. a) Are you currently living in your own home? Yes  (1) No  (2)

b) *If* ***no****, who are you staying with? ..........................................................................*

c) *If* ***no****, why have you moved? .................................................................................*

12. Housing **at time of interview**

Rented house  (1)

Owned house  (2)

Rented apartment  (3)

Owned apartment  (4)

Staff quarter  (5)

Others  (6)

Others, specify……………………………………………….

13. Housing **before stroke?**

Rented house  (1)

Owned house  (2)

Rented apartment  (3)

Owned apartment  (4)

Staff quarter  (5)

Others  (6)

Others specify…………………………………………………………………..

14. Marital status **now**?

Married, living together  (1)

Married, not living together  (2)

Single  (3)

Widow/Widower  (4)

Divorced/separated  (5)

15. Marital status **before stroke?**

Married, living together  (1)

Married, not living together  (2)

Single  (3)

Widow/Widower  (4)

Divorced/separated  (5)

1. How many members do you have in your household (including the respondent)?...............

16. a) How many biological children do you have? ...........................................................

b) How many in the household are totally economically dependent? ….......................

c) How many in the household are partly economically dependent?­­­................................

d) How many children (both biological and extended) below the age of 18 are living at

home?.........................

e) Do you have access to a mobile phone? Yes  (1) No  (2)

**Clinical Characteristics**

17. How old were you at stroke onset? ………………years

18. Type of stroke *(if information from………………………….)*

Ischemic stroke  (1)

Haemorrhagic stroke  (2)

Other  (3)

Specify other ...........................................................................

19. CT scan *(if information from…………………………………….)*

Yes  (1) No  (2)

20. What side of the brain, which hemisphere is affected? *(if information…………………)*

Right  (1)

Left  (2)

Others  Specify:.................................

1. a) Have you received any rehabilitation? Yes  (1) No  (2)

b) If yes, what rehabilitation intervention/-s have you received?……………

………………………………………………………………………………………

22. Have you received any medical health care (nurse/physician) for stroke?

Yes  (1) No  (2)

a) *If* ***yes,*** *where?* ………………………………..

1. Have you received any medical rehabilitation? Yes  (1) No  (2)
2. *If* ***yes****, where* *……………………………..*

b) *If* ***yes****, what rehabilitation intervention/-s have you received?*

(1) Occupational therapy

(2) Physiotherapy

(3) Language and speech therapy

(4) Other

c) *If* ***other****, what have you received? ..........................................................................................*

1. Have you received any assistance from a healer? Yes  (1) No  (2)
2. *If* ***yes****, which assistance did you get?*  (1) Herbal medicine

(2) Spiritual healing (from religious person)

(3) Traditional healing (from witchdoctor)

1. How do you describe the state of your health **now**?

Very good  (1)

Good  (2)

Fair  (3)

Bad  (4)

Very bad  (5)

1. How do you describe the state of your health as compared to your friends of your age **now**? Do you feel it is

Much better  (1)

Little better  (2

The same  (3)

A little worse  (4)

Much worse  (5)

27. Were you mentally or physically incapacitated in any way **before the stroke?**

Yes  (1) No  (2)

28. Did you get any help in your daily activities from someone before the stroke?

Yes  (1) No  (2)

*If* ***yes****, from whom?* Family member  (1)

Neighbour  (2)

Friend  (3)

Other  (4)

29. a) Do you get any help in your daily activities from someone **now?**

Yes  (1) No  (2)

*If* ***yes****, from whom?* Family member  (1)

Neighbour  (2)

Friend  (3)

Other  (4)

b) *If* ***yes****, how often do you get help*? Daily  (1) Weekly  (2)

c) Which activities do you find difficult?.......................................................... ……….

...........................................................................................................................................

…………………………………………………………………………………………

30. Did you use assistive aids **before stroke?** Yes  (1) No  (2)

1. *If* ***yes****, which assistive aids did you use?*

Crutch  (1)

Cane/stick  (2)

Walker  (3)

Wheelchair  (4)

Other  (5)

Other, specify………………………………………………

31. Do you use assistive aids **now?** Yes  (1) No  (2)

1. If yes, which assistive aids do you use? (tell the respondent you want to see it)

Crutch  (1)

Cane/stick  (2)

Walker  (3)

Wheelchair  (4)

Other  (5)

Other, specify…………………………………………

32. **Are you a member of Stroke Association of Kenya? Yes** (1) **No** (2)

33. **How did you got in contact with Stroke Association of Kenya?**

Hospital  (1) which hospital ___________________

Friend  (2)

Family member  (3)

Rehabilitation centre  (4) which centre______________________

Public media News etc  (5)

Internet  (6)

Other  (7) how_____________________________

34. Why do you think it is important?­­­ Give one example

___________________________________________

35. What do you think that you and the association could do? Give one example

__________________________________________________

37. When should someone become a member? Give one example

**________________________________________________________**
