# Supplementary material for: Living with consequences of stroke and risk factors for unhealthy diet- experiences among stroke survivors and caregivers in Nairobi, Kenya
Source: BMC Public Health. 2021 Mar 16;21:511. doi: 10.1186/s12889-021-10522-4 (PMC7968355; doi:10.1186/s12889-021-10522-4)
Supplement: Supplementary file 2 — Additional file 2: Supplementary material 2. The second round of data collection Description of data included in the file: The interview protocol. Not included in the manuscript. [file 12889_2021_10522_MOESM2_ESM.docx]

***Supplementary material 2***

**Stroke rehabilitation project, Data collection 2.**

**INTERVIEW PROTOCOL**

**To describe**

**1) the pathway from getting a stroke to now**

**and 2) the experiences of consequences of stroke of the person who have had a stroke**

**and 3) what nutritional guidance has been given, nutrition status and food habits**

**Height (cm) Weight (kg)**

**Waist circumference (cm) BMI**

***Follow-up from first data collection (Nov -18)***

1. Are you currently living in your own home? Yes (1) No (2)

*If* ***no****, who are you staying with? why have you moved?*

2. Housing **at time of interview:** rented ho, owned ho, rented ap, owned ap, staff quarter, others?

3. How many members are there in your household (including the respondent)?

4. How many biological children do you have?

How many in the household are totally economically dependent­?

How many children (both biological and extended) below the age of 18 are living at home?

5. What rehabilitation intervention/-s have you received?

(1) Occupational therapy (2) Physiotherapy (3) Language and speech therapy (4) Other

6. Have you received any assistance from a healer? Yes (1) No (2)

*If* ***yes****, which assistance did you get?* Herbal medicine

Spiritual healing (from religious person)

Traditional healing (from witchdoctor)

7. Were you mentally or physically incapacitated in any way **before the stroke?**

***Open questions for person with stroke:***

1. Tell me about yourself?

***Probe for****; Age, marital status, what do you do for income, religion?*

2. Tell me about your health? (objective 1)

***Probe for*** *stroke, when it happened*, *what did you do to address /solve this problem? When it occurred, how did you feel when it occurred, how did you come to know that it was stroke, what did you do to address the problem,*

***Probe for*** *the first point of contact for the care sought, types of care you sought/biomedical/traditional/herbal/spiritual, how did you feel about your health/stroke after seeking care?*

3. In the last 7 days, many times a day do you usually have a meal? (objective 3)

4. Since suffering the stroke, have your eating habits changed in any way? (objective 3)

**Probe for:** possible change, reason for this change (physical limitations due to stroke;

depression; loss of income etc). Establish how eating habits looked before stroke and today.

5. Follow up questions depending on answer. If other categories

- 1. Physical limitations (e.g. difficulty feeding oneself; dysphagia).

**Probe for:** specifically, how eating has been affected; does this affect the household; does the person get help with preparing food and feeding

- 1. Changes in psychological state

**Probe for:** How does this affect the person and the household.

- 1. What support is given?
  2. Loss of income.

**Probe for:** How has this affected eating habits and grocery shopping. How has this affected the household.

6. What help/support in terms of cooking and eating did you get while seeking care? (objective 1)

***Probe for*** *who helped, i.e. practicing professional, family/relative/neighbour, kind of help offered, when did it stop?*

7. What challenges did you experience while seeking the care mentioned above? (objective 1)

***Probe*** *problems faced at individual and household levels?*

8. Tell me about your how your day to day activities was like before the stroke? (objective 2)

*Focus on grocery shopping, cooking etc*

9. Tell me about your day to day activities today? (objective 2)

***Probe for*** *a how a typical day look like? Probe for support offered to execute these activities. Changes before/ after stroke?* *Focus on grocery shopping, cooking*

10. Tell me about what is important to you in your life today? (objective 2)

11. Tell me if you miss doing something that you did before you got a stroke? (objective 2)

12. Would you agree that what you eat, and drink can affect your physical well-being?

***Probe for*** *awareness of how lifestyle affects chances of suffering from NCDs*
